# The Effect of Different Disinfecting Agents on Bond Strength of Resin Composites

**DOI:** 10.1155/2014/231235

**Published:** 2014-11-13

**Authors:** Ahmed Mohammed Hassan, Ahmed Ali Goda, Kusai Baroudi

**Affiliations:** ^1^Al-Farabi College, King Abdullah Road East, Ishbilia, P.O. Box 85184, Riyadh 11691, Saudi Arabia; ^2^Department of Operative Dentistry, Al-Azhar University, Assiut, Egypt

## Abstract

*Objective*. The aim of this study was to evaluate the effect of different disinfectant agents on bond strength of two types of resin composite materials. *Methods*. A total of 80 sound posterior teeth were used. They were divided into four groups (*n* = 20) according to the dentin surface pretreatment (no treatment, chlorhexidine gluconate 2%, sodium hypochlorite 4%, and EDTA 19%). Each group was divided into two subgroups according to the type of adhesive (prime and bond 2.1 and Adper easy one). Each subgroup was further divided into two subgroups according to the type of resin composite (TPH spectrum and Tetric EvoCeram). Shear bond strength between dentin and resin composite was measured using Universal Testing Machine. Data collected were statistically analyzed by *t*-test and one-way ANOVA followed by Tukey's *post hoc* test. *Results*. It was found that dentin treated with EDTA recorded the highest shear bond strength values followed by sodium hypochlorite and then chlorhexidine groups while the control group showed the lowest shear bond strength. *Conclusions*. The surface treatment of dentin before bonding application has a great effect on shear bond strength between resin composite and dentin surface.

## 1. Introduction

Restoring posterior teeth with resin-based composite materials continues to gain popularity among clinicians, and the demand for such aesthetic restorations is increasing. Indeed, the most common aesthetic alternative to dental amalgam is resin composite [1]. Long-term studies have shown that the bond strength of resin-bonded dentin decreased over time due to collagen degradation within the hybrid layer [2, 3]. Meiers and Shook 1996 indicated that residual bacteria might proliferate from the smear layer beneath restorations [4]. Therefore, the adjunctive use of antibacterial solutions after cavity preparation may be considered a method to reduce the incidence of postoperative sensitivity by eliminating viable bacteria and their toxins from the restoration-tooth interface [5].

Chlorhexidine (CHX) is widely used as an antimicrobial agent for disinfection before placement of restorations. Loss of hybrid layer integrity compromises resin-dentin bond stability. Matrix metalloproteinase (MMP) may be partially responsible for hybrid layer degradation. CHX acts as matrix metalloproteinase (MMP) inhibitor, so it has beneficial effects on the preservation of dentin bond strength. CHX also minimizes the convective and evaporative water fluxes from the underlying dentin, thus enhancing the bonding capacity of the self-etch adhesive [6]. Sodium hypochlorite (NaOCl) is widely used as chemomechanical caries removal and in dentin bonding techniques, because of its antimicrobial and tissue dissolving properties [7]. Since the smear layer composition is similar to the originating tissue (50 volume % mineral and 30 volume % collagen), the application of (NaOCl) over the smear layer covered dentin would eliminate its collagen phase resulting in reduction in the smear layer compactness. This property enhanced the bonding of the self-etching adhesive as it might increase the diffusively of the acidic monomers, through water-filled channels between particles of smear layer enlarging them to reach and interact with the underlying dentin surface [6]. EDTA is a weak acid and has a disinfectant and demineralizing effect. It has been widely used to dissolve the mineral phase of dentin without altering the structure of dentin collagen [8].

The purpose of this study was to evaluate the effect of disinfectant agent on shear bond strength between dentin and two types of resin composite and to evaluate the failure pattern. The null hypothesis was that disinfectant agent has no effect on bond strength between resin composites and dentin.

## 2. Materials and Methods

Eighty molars were used in this study; a prior patient's consent was obtained. Approval of Al-Azhar University, Faculty of Oral and Dental Medicine, Egypt (under number 456/2013), was also obtained. The inclusion criteria included teeth that needed to be extracted due to periodontitis, pericoronitis, and unerupted or impacted teeth. The exclusion criteria included teeth that were decayed or damaged during the extraction and also those teeth that were congenitally affected such as enamel hypoplasia or amelogenesis/dentinogenesis imperfecta. Once the teeth were extracted, they were stored in distilled water at 4°C and used within two months following extraction. Before the study, all teeth were scaled and cleaned using pumice and rubber cups.

The teeth, including the roots, were embedded inside a cylindrical-shaped mold filled with self-cured acrylic resin (Acrostone Dental Factor, UK) till the cervical line with the occlusal plane being parallel to the floor. After completing the polymerization of the acrylic resin, the tooth in the set acrylic resin was removed from the mold and the occlusal enamel of the teeth was removed perpendicular to the long axis of teeth with a low-speed diamond disk saw (IsoMet; Buehler, Lake Bluff, IL, USA) and then fissure bur was used to complete the preparation until 1 mm beyond the dentinoenamel junction.

The specimens were divided into four main groups: A (*n* = 20), according to the proposed dentin surface pretreatment:

(A_1_) a control group without pretreatment;
(A_2_) pretreatment with chlorhexidine gluconate 2% (Consepsis, Ultradent, USA);
(A_3_) pretreatment with NaOCl 4% (central drug house (p), New Delhi, India**);**
(A_4_) pretreatment with EDTA 19% (File-Eze, Ultradent, USA).

The disinfectant in every group was applied using a disposable brush tip, left undisturbed for 20 seconds, then rinsed with water for 10 seconds, and dried with absorbent paper. Each main group was divided into two subgroups (*n* = 10) according to type of adhesive system:

(B1) etch and rinse;
(B2) self-etch adhesive.

For subgroup (B1) the dentin surface of each specimen was etched with 37% phosphoric acid (Condicionador, Dentsply, Brazil) for 15 seconds, rinsed with water for 20 seconds, and dried with absorbent paper. Then the self-priming adhesive (Prime & Bond 2.1, Dentsply, Brazil) was applied using a fully saturated brush tip and lightly air-dried for 5 seconds and light-cured for 20 seconds with halogen light curing unit (Cromalux-E mega-physics dental Rastatt, Germany) with a light output of 600 mW/cm^2^. For subgroup (B2) (Adper easy one 3 M- ESPE, AG Seefeld, Germany) self-etching adhesive was applied and left undisturbed for 20 seconds, lightly air-thinned for 5 seconds, and light-cured for 20 seconds by the same curing unit. Each subgroup was further divided into two subgroups (*n* = 5) according to the type of resin composite:

(C1) microhybrid resin composite (TPH, Dentsply, Brazil);
(C2) nanohybrid resin composite (Tetric Evoceram, Ivoclar-Vivadent, Schaan, Liechtenstein).

Either type of composite was carefully applied to the treated dentin surface by placing the material into cylindrical-shaped split Teflon mold with an internal diameter of 3 mm and a height of 3 mm. Composite was placed incrementally in 2 layers, 1.5 mm each; each layer was light-cured for 20 seconds with the previous light curing unit.

**Figure 1:**
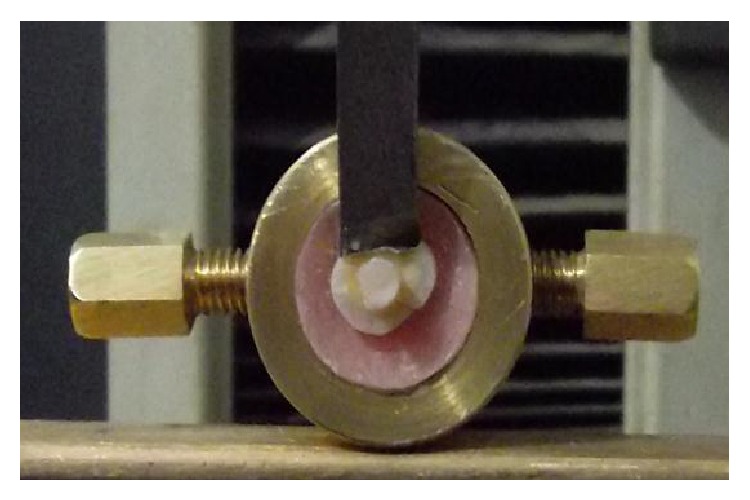
Sample secured to the lower fixed compartment of testing machine by tightening screws.

A circular interface shear test was designed to evaluate the bond strength. All samples were individually and horizontally mounted on a computer controlled materials testing machine (Model LRX-plus; Lloyd Instruments Ltd., Fare ham, UK) with a load cell of 5 kN and data were recorded using computer software (Hexogen-MT; Lloyd Instruments). Samples were secured to the lower fixed compartment of testing machine by tightening screws (Figure 1). Shearing test was performed by compressive mode of load applied at resin-tooth interface using a mono-beveled chisel shaped metallic rod attached to the upper movable compartment of testing machine traveling at cross-head speed of 0.5 mm/min.

Both surfaces of each fractured specimen were examined using USB digital microscope (Scope Capture Digital Microscope, Guangdong, China) at 30x magnification and were photographed using image analysis software (Scope Capture 1.1.1.1. Ltd.) in order to determine the mode of failure.

*Statistical Analysis*. One-way ANOVA followed by Tukey's* post-hoc* test were performed to detect significance between groups. Independent* t*-test was performed to detect significance between subgroups. All statistical analysis was conducted at the significance level of 0.05.

## 3. Results

**Figure 2:**
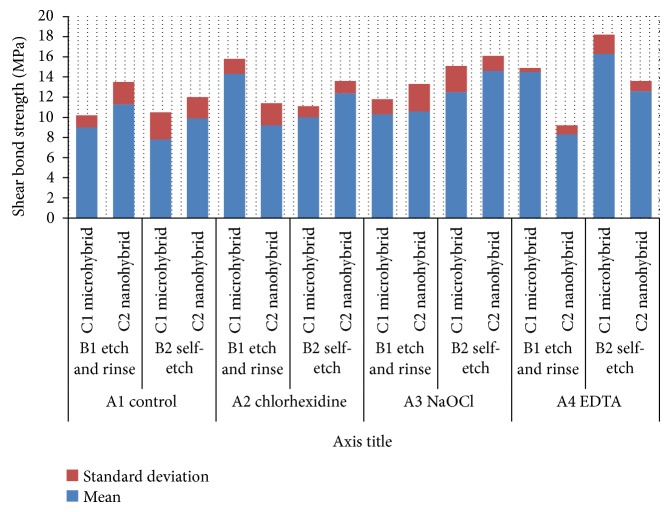
Mean shear bond strength (MPa) for all groups.

Mean values and standard deviations of all groups are shown in Figure 2. For the control group, the highest mean shear bond strength (11.3 ± 2.2 MPa) was recorded for nanohybrid composite bonded to dentin specimen using etch-and-rinse adhesive, while the lowest mean shear bond strength (7.8 ± 2.7 MPa) was recorded for microhybrid composite bonded to dentin specimen using self-etch adhesive. Shear bond strength for specimens treated with chlorhexidine ranged from (9.2 ± 2.2 MPa) for nanohybrid composite bonded to dentin using etch-and-rinse adhesive to (14.3 ± 1.5 MPa) for microhybrid composite bonded to dentin using etch-and-rinse adhesive. For (NaOCl) group, specimens treated with NaOCl and bonded to nanohybrid composite resin using self-etch adhesive showed the highest mean shear bond strength (14.6 ± 1.5 MPa) while those bonded to microhybrid composite using etch-and-rinse adhesive recorded the lowest mean shear bond strength (10.3 ± 1.5). For EDTA group, microhybrid composite bonded to dentin specimens using self-etch adhesive showed the highest mean shear bond strength (16.3 ± 1.9 MPa), while the lowest mean shear bond strength (8.3 ± 0.9) was recorded for those specimens bonded to nanohybrid composite using etch-and-rinse adhesive.

**Figure 3:**
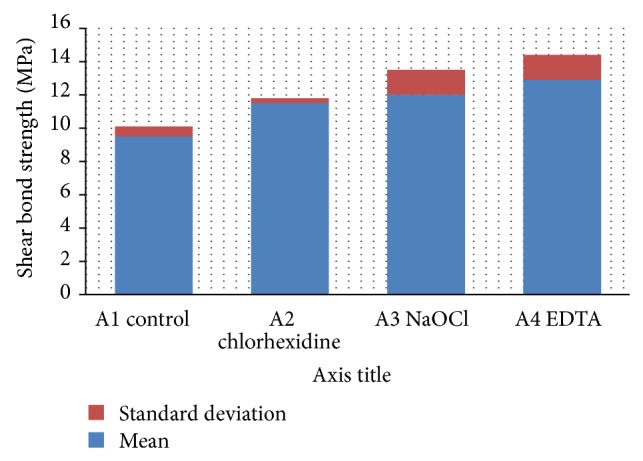
The effect of different surface pretreatments on the mean shear bond strength values.

Regardless of composite type or bonding agent, totally it was found that EDTA treated dentin recorded the highest statistically significant (*P* < 0.05) mean shear bond strength (12.9 ± 1.5 MPa) followed by NaOCl treated dentin (12 ± 1.5 MPa) and then chlorhexidine treated dentin (11.5 ± 0.3 MPa), while the control group showed the lowest statistically significant shear bond strength (9.5 ± 0.6 MPa) (Figure 3).

**Figure 4:**
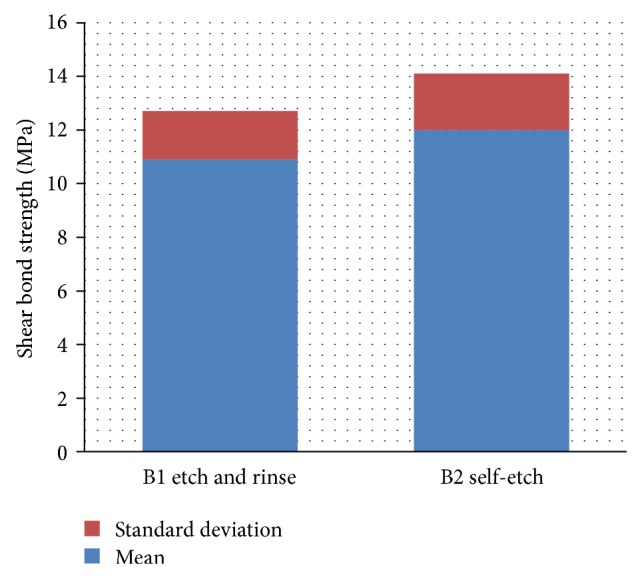
The effect of the adhesive system on the shear bond strength values.

Regardless of composite or disinfectant, it was found that self-etch bonding agent recorded higher shear bond strength mean value (12 ± 2.1 MPa) than total etch bonding agent (10.9 ± 1.8 MPa) (Figure 4). This difference was statistically significant (*P* < 0.05).

**Figure 5:**
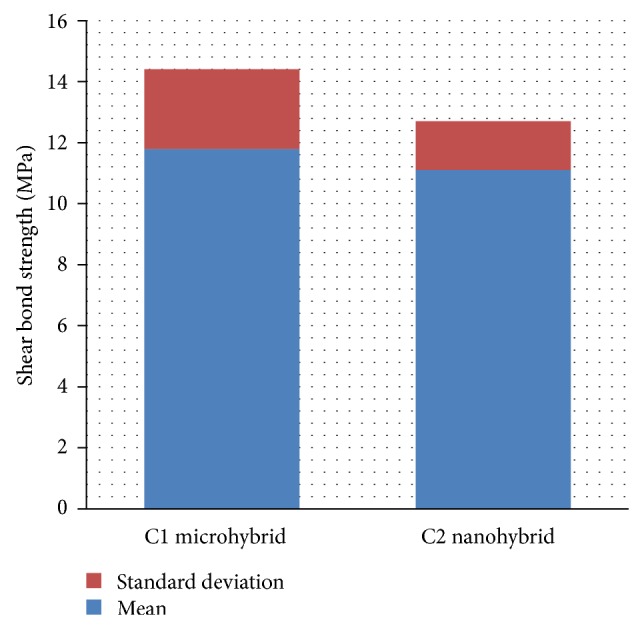
The effect of the composite type on the shear bond strength values.

Regardless of disinfectant or bonding agent, it was found that microhybrid composite recorded higher shear bond strength mean value (11.8 ± 2.6 MPa) than nanohybrid composite (11.1 ± 1.6 MPa) (Figure 5). This difference was statistically not significant (*P* > 0.05).

Regarding mode of failure, adhesive mode of failure represented 80% with 20% mixed failure in the control group (no pretreatment) with self-etch adhesive using microhybrid composites, chlorhexidine group with etch-and-rinse adhesive using nanohybrid composite, EDTA group with etch-and-rinse adhesive using both microhybrid and nanohybrid composites, and with self-etch adhesive using nanohybrid composite. Adhesive mode of failure represented 60% with 40% mixed failure in chlorhexidine group with etch-and-rinse adhesive using microhybrid composite, NaOCl group with etch-and-rinse adhesive using both microhybrid and nanohybrid composites, and with self-etch adhesive using nanohybrid composite.

**Figure 6:**
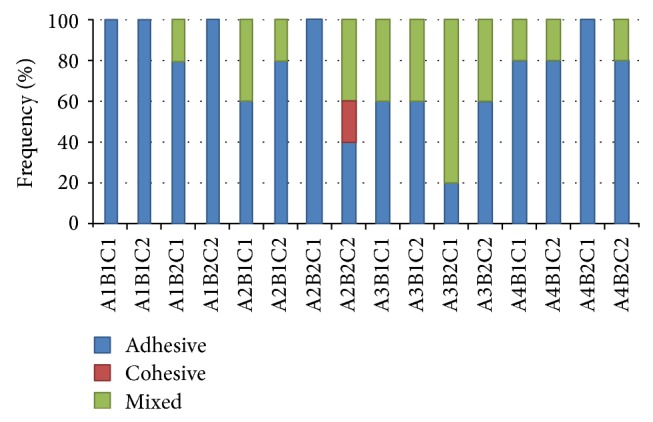
Different failure modes for all groups.

**Figure 7:**
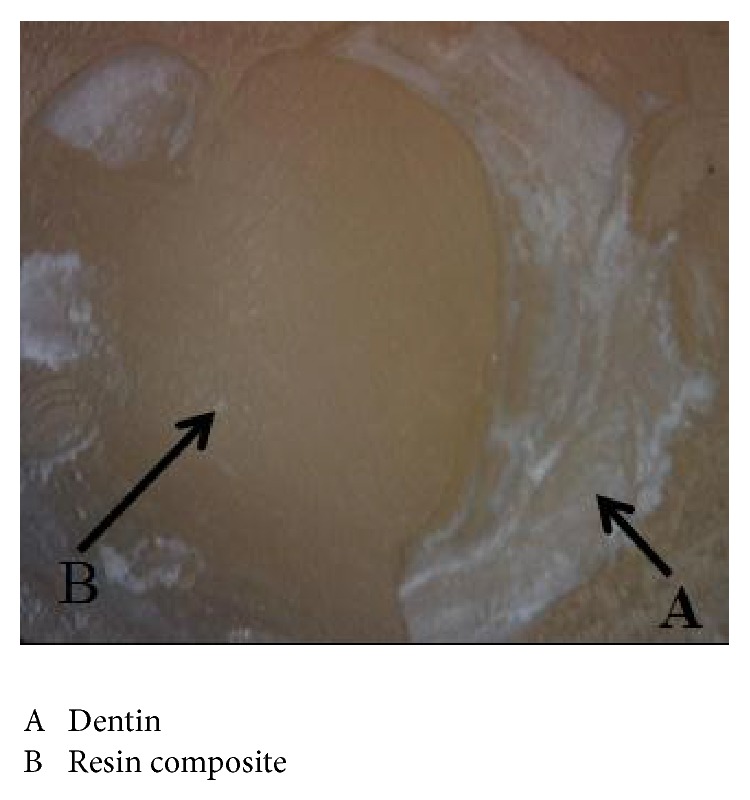
Mixed failure mode.

However, adhesive failure represented only 20% with 80% mixed failure only in NaOCl group with self-etch adhesive using microhybrid composite. Furthermore, cohesive failure only demonstrated 20% in chlorhexidine group with self-etch adhesive using nanohybrid composite. This group also demonstrated 40% adhesive and 40% mixed failure. Failure mode percentages of all groups are illustrated in Figure 6 and mixed failure mode is shown in Figure 7.

## 4. Discussion

Regardless of composite type or bonding agent, it was found that EDTA treated dentin recorded the highest shear bond strength followed by NaOCl treated dentin and then chlorhexidine treated dentin while the control group showed the lowest shear bond strength. Our result is in agreement with previous studies [9–11] which attributed the improvement in bond strength to the removal of the smear layer, which prevents direct contact of the self-etching adhesive with dentin; consequently, removal of the smear layer facilitates the formation of a stronger and more homogeneous hybrid layer, while other studies found that treatment of dentin with EDTA produced no significant difference in bond strength compared to that produced with groups which were etched with phosphoric acid [12, 13]. This disagreement may be attributed to their use of EDTA as etching material instead of the phosphoric acid, so they used EDTA in high concentration for long durations while in the current study EDTA was used as a cavity disinfectant in lower concentration before dentin etching.

Sodium hypochlorite application prior to acid etching significantly increased the bond strength of both adhesive systems used. This result is in agreement with the result of previous studies [14, 15]. They attributed the increase bond strength to the elimination of collagen layer which was removed by application of NaOCl leading to a better penetration of the adhesive into intertubular dentin. This increase in bond strength may be also due to removal of smear layer by NaOCl. Complete removal of smear layer might enhance the bonding to dentin as it facilitates the penetration of resin monomer leading to complete infiltration of the demineralized layer by numerous resin tags. On the other hand, it was reported that sodium hypochlorite significantly decreased the bond strength to dentin [16], which is in contrast to our results. They showed that NaOCl damages the organic component of dentin; therefore, organic monomers do not sufficiently penetrate into the demineralized dentin, resulting in a lack of proper bond strength, while another study reported that sodium hypochlorite does not influence the bond strength to dentin [17]. The disagreement of the result of those studies with the present study may be attributed to differences in sample preparation methods, application mode, and time. In this study 4% sodium hypochlorite was used for 20 sec prior to dentin etching, while the previous studies used NaOCl in different concentrations after dentin etching.

Chlorhexidine treated dentin had higher shear bond strength than control group. These results are inconsistent with certain studies which showed that a CHX cavity disinfectant had an adverse effect and produced significantly lower bond strengths [18, 19]. On the other hand, some studies reported that CHX had no influence on the shear bond strength to dentin [20, 21]. The disagreement in the results of those studies with the present study may be attributed to differences in modes of use of CHX: before etching, after etching, rinsing off, or not rinsing, also the form of material (gel or solution) and time of application. Using of a CHX before etching was shown to not to affect bonding to dentin, however, reduced dentin bond strengths usually when a CHX was used after etching, but rinsing the CHX off before bonding produced bond strengths that were similar to no-cleanser controls [22]. Rinsing away CHX prior to bonding will most likely prevent undesired material interactions.

Among several factors that may interfere with the quality of bonding, the type of adhesive systems used is of great importance.

It was found that etch-and-rinse adhesive recorded statistically nonsignificant higher shear bond strength mean value than self-etching adhesive. The obtained data is consistent with previous studies which reported that the dentin bond strength of self-etching adhesives was comparable to that of the etch-and-rinse systems [14, 23]. One of the advantages of self-etching adhesives is that dentin conditioning and priming occur simultaneously, resulting in the formation of a strong void-free hybrid layer [11], while other studies [24, 25] found that etch-and-rinse adhesive showed higher bond strength than self-etch adhesives. In contrast, Giriyappa and Chandra, 2008, showed that the self-etching primer had higher mean shear bond strength than total etch adhesive [26].

In this study, groups treated with disinfectants recorded statistically significant higher shear bond strength for self-etch bonding agent than etch-and-rinse bonding agent. Since self-etching adhesives have higher pH values than the phosphoric acid used and are not rinsed away, the smear layer or its components are incorporated into the bonded layers [27]. For strong self-etching adhesives, the smear layer and smear plugs should be dissolved to overcome the main problems during using self-etching adhesives. So in the current study, the increased bond strength of self-etch was attributed to removal of the smear layer and smear plugs by the effect of used EDTA, NaOCl, or CHX.

The lowest shear bond strength was recorded for microhybrid composite bonded to dentin specimens with self-etch adhesive without any pretreatment (control), while the highest shear bond strength in the study was for microhybrid composite bonded to dentin specimen with self-etch adhesive treated with EDTA. This may be attributed to the self-etch which has the problem of the smear layer and smear plugs that interfere with bonding [27]. The effect of dentin pretreatment with EDTA on shear bond strength of the other group may be due to the complete removal of smear layer. However, bond strength is multifactorial in nature, having many variables affecting it. Therefore, further studies might be of importance in determining the effect of using EDTA, sodium hypochlorite, or CHX prior to the application of the different adhesives in the market.

All groups showed percentage of adhesive failures but we observed that the failure mode was predominantly adhesive for control group with increased percentage of mixed failure for groups of disinfectants. This result is in agreement with other studies [18, 28, 29]. On the other hand, our result is in disagreement with the result of another study [17], because the failure mode was predominantly mixed. In control groups, there was no difference between etch-and-rinse adhesive and self-etch adhesive, which is in accordance with certain studies which found that failure mode of both adhesives was mostly adhesive [30]. The increased percentage of mixed failure on groups of disinfectants was attributed to the increased shear bond strength which clearly was reflected by the mode of failure of the bonding system. This is in agreement with the study of Ceballos et al., 2003. They reported that the major mode of failure in specimens with low bond strengths was adhesive failure, while cohesive fractures in dentin or composite were seen at higher bond strength [25].

## 5. Conclusion

The surface treatment of dentin before bonding positively affects the shear bond strength between resin composite and dentin especially with self-etch adhesive. The type of resin composite used affects the recoded shear bond strength values.
